# The Origins of Passive, Active, and Sleep-Related Fatigue

**DOI:** 10.3389/fnrgo.2021.765322

**Published:** 2021-12-23

**Authors:** Steven D. Chong, Carryl L. Baldwin

**Affiliations:** Department of Psychology, Program of Human Factors Psychology, Wichita State University, Wichita, KS, United States

**Keywords:** fatigue, arousal, circadian rhythm, vigilance, driving, automation, attention, vigilance decrement

## Abstract

Driving is a safety-critical task that requires an alert and vigilant driver. Most research on the topic of vigilance has focused on its proximate causes, namely low arousal and resource expenditure. The present article aims to build upon previous work by discussing the ultimate causes, or the processes that tend to precede low arousal and resource expenditure. The authors review different aspects of fatigue that contribute to a loss of vigilance and how they tend to occur; specifically, the neurochemistry of passive fatigue, the electrophysiology of active fatigue, and the chronobiology of sleep-related fatigue.

## Introduction

According to the Centers for Disease Control Prevention (2020), motor vehicle crashes are the second leading cause of accidental or unintentional death in the United States. Driver distraction and drowsiness are thought to be the most common causes for roadside crashes (AAA Foundation for Traffic Safety, 2018). Driving automation systems (DASs; e.g., adaptive cruise control and active lane keeping) have been introduced to mitigate these crash rates by reducing workload on the driver (Wickens et al., 2010; Wickens, 2018). They may, however, inadvertently introduce new problems to the driver (Mueller et al., 2021), such as increasing the prevalence of driver distraction (Young, 2012; Greenlee et al., 2018), drowsiness (Gimeno et al., 2006; Gaspar et al., 2017; Sikander and Anwar, 2018; Kundinger et al., 2020), engagement in non-driving related tasks (Seppelt and Victor, 2016; Cabrall et al., 2019; Mueller et al., 2021), and loss of situational awareness (Berberian et al., 2017; Brandenburg and Chuang, 2019; Lohani et al., 2019). Overestimating the capabilities of DASs can also lead to overreliance and complacency in the system as well, which may further exacerbate the aforementioned consequences (Parasuraman et al., 2000; Schaefer et al., 2016; Seppelt and Victor, 2016; Hecht et al., 2018). Detailed examination into the processes underlying distraction and sleepiness—or fatigue, more broadly—will be critical to maintaining roadway safety in this time of increasing prevalence of DASs. Here, the authors define fatigue as an adaptive state of stress that occurs due to the interaction between an individual and their environment (Hancock and Warm, 1989). The goal of the present article is to build upon the work of May and Baldwin (2009) by providing more in-depth information on the causality of fatigue, given the findings of newer research. We begin our examination by first discussing a common indicator of fatigue, the vigilance decrement. Then, the remainder of the article will discuss the underlying mechanisms of three dimensions of fatigue: passive, active, and sleep-related fatigue.

The authors want to quickly note that driving using automation will be the primary example used throughout the present paper, however the underlying mechanisms discussed below may also be applicable to other tasks and domains that involve vigilance as well, such as radar operators, anesthesia monitors, air traffic controllers, and cockpit pilots (Donald, 2008; Wiggins, 2011).

## The Vigilance Decrement

Berberian et al. (2017) identified the vigilance decrement as one of the critical factors that impacts a driver's situational awareness when using automation technologies. The vigilance decrement can be defined as “[t]he deterioration in human performance resulting from adverse working conditions…” (Mackworth, 1948, p. 6). Researchers have studied various aspects that contribute to the aforementioned “adverse working conditions,” such as prolonged time-on-task (Mackworth, 1948; Langner and Eickhoff, 2013; Thomson et al., 2015), under-stimulating task features (Scerbo, 1998; Greenlee et al., 2018; Luna et al., 2021), degrading goal maintenance (Hockey, 2011; Braver, 2012; Grahn and Manly, 2012), low critical-event rate (Parasuraman et al., 1987; Langner and Eickhoff, 2013), poor display usability (Hancock, 2013, 2017), and the need to maintain high workload (Helton and Russell, 2017). During a vigilance decrement, one's cognitive state tends to be vulnerable to both distraction (Aston-Jones and Cohen, 2005; Greenlee et al., 2018) and drowsiness (Dinges, 1995; Thiffault and Bergeron, 2003; de Naurois et al., 2019). In this compromised state, a driver is likely to disengage from attentive driving, or monitoring, and instead engage in non-driving related tasks (Randall et al., 2014; Körber et al., 2015; Greenlee et al., 2018).

Most research on vigilance, at least within the Human Factors domain, has focused on the variables and conditions that tend to correlate or precede the vigilance decrement, such as low arousal (Scerbo, 1998), resource depletion (Warm et al., 1996), and neural activity (Smallwood et al., 2012b). Based on this research, two fundamental theories have emerged: the underload, or mindlessness, theory (Sawin and Scerbo, 1995; Scerbo, 1998; Manly et al., 1999) and the overload, or resource depletion, theory (Warm et al., 1996). The underload theory posits that vigilance tasks induce boredom due to their intrinsically monotonous, under-stimulating, and infrequently responding nature. Individuals may, therefore, engage in other activities, such as mind-wandering, in order to elevate their arousal, thereby alleviating boredom (Seli et al., 2015).

The overload theory, on the other hand, suggests that vigilance tasks are inherently difficult due to the high workload associated with having to maintain attention over a prolonged period of time. Specifically, cognitive resources are thought to be allocated toward maintaining proper executive control during a vigil, and once those resources have been exhausted, performance detriments ensue (Randall et al., 2014). Although some have suggested that the under- and overload theories are antithetical to each other, they may describe different dimensions of fatigue.

This has led researchers to adopt an altered perspective regarding the claims made by both underload and overload theories. For instance, some (e.g., Pattyn et al., 2008; Langner and Eickhoff, 2013) view the former as describing the vigilance decrement from an exogenous, “bottom-up” perspective (e.g., monotonous and understimulating task characteristics) and the latter from an endogenous, “top-down” perspective (e.g., subpar executive functioning due to depleted cognitive resources). Similarly, others (e.g., Gimeno et al., 2006; May and Baldwin, 2009; Di Stasi et al., 2012) suggest that these theories describe different aspects of fatigue, passive and active fatigue, respectively. Both underload and overload theories alone, however, only describe the proximate causes of the vigilance decrement, not the underlying, ultimate causes. In other words, low arousal and resource depletion both tend to precede the occurrence of fatigue, but this leads to the question, what precedes low arousal and resource depletion?

## Processes Underlying Passive Fatigue

In the following section, the role of the locus coeruleus and norepinephrine (LC-NE) system will be discussed as it relates to arousal: specifically, the way in which it modulates its firing rate to either broaden or narrow our attentional filter, how it recruits different brain regions to assess the costs and benefits of performing a goal-directed task, and how it works with other brain networks to facilitate exploitative or explorative behavior.

The LC-NE system is one of the primary brainstem neuromodulatory systems that influences arousal (Sara and Bouret, 2012). The locus coeruleus (LC), having broad afferent and efferent connections, is responsible for almost all of the norepinephrine (NE) activity in the neocortex, which regulates the excitatory and inhibitory effects of postsynaptic neurons associated with information selection and processing (Aston-Jones and Cohen, 2005; Bouret and Sara, 2005; Sara and Bouret, 2012). Aston-Jones and Cohen (2005) argued that the LC-NE optimizes reward-contingent behavior by modulating arousal levels such that it either broadens or narrows our attentional filter. The LC-NE either broadens attention in search for a more rewarding task, or it narrows attention to prevent distractions. The LC has two aspects to its firing rate, tonic and phasic. Tonic firing refers to the baseline firing rate and is thought to be indicative of one's current arousal state, while phasic firing refers to the changes in firing in response to task-relevant stimuli presentation and is thought to reflect the degree of cognitive processing (Murphy et al., 2011; Joshi and Gold, 2020). Tonic firing rate, as it relates to performance on a task, resembles the Yerkes-Dodson curve (Yerkes and Dodson, 1908). When the LC exhibits a non-optimal tonic firing rate that is either too high or too low, there are broader neuronal responses to sensory stimuli, which in turn promotes distractibility as it blends the saliency of task-relevant and task-irrelevant stimuli (Mittner et al., 2014, 2016). When the LC exhibits a moderate tonic firing rate, the signal-to noise ratio of phasic responses become salient, which in turn facilitates the discrimination between relevant and irrelevant stimuli, thus promoting sustained attention toward the primary task (Bouret and Sara, 2005).

The firing rate of the LC-NE system has been commonly studied by observing changes in pupillometry due to the strong correlations among LC-NE activity, pupillary dynamics, and arousal state (Mittner et al., 2014, 2016; Hopstaken et al., 2015; Lohani et al., 2019; McWilliams and Ward, 2021). For instance, in a series of experiments, Unsworth and Robison (2018) found that smaller pretrial pupil sizes (as an index of tonic firing) and smaller task-evoked pupillary responses (as an index of phasic firing) were related to lower arousal, poorer performance, and more task-unrelated thoughts. Similarly, Körber et al. (2015) found that passive fatigue was induced when participants were monitoring a driving simulator with automation capabilities that controlled longitudinal and lateral steering, as indexed by a continual overall decrease in pupil diameter, greater reports of mind-wandering at the end of the monitoring task, and a general trend of slower reaction times on an auditory oddball task. Although the functional and anatomical mechanisms are not yet fully understood [but see Joshi and Gold (2020), Murphy et al. (2014)], previous research suggests that a task's utility—or the costs and benefits associated with performance on a task—may dictate the firing rate of the LC.

The anterior cingulate cortex (ACC) and the orbitofrontal cortex (OFC) project information regarding a task's utility to the LC-NE system (Aston-Jones and Cohen, 2005). As the costs associated with a task increase—as evaluated by the ACC—the LC exhibits high tonic firing for the search of alternative forms of reward, and it promotes engagement in other tasks (e.g., mind-wandering). On the other hand, as the benefits outweigh the costs—as evaluated by the OFC—the LC exhibits moderate tonic firing, which in turn helps prevent attention from being oriented to task-irrelevant stimuli. The ACC and OFC, receiving inputs from various somatosensory and limbic structures, evaluate a task's utility both in the short- and long-term.

During initial engagement of a goal-directed task, reinforcement learning occurs to favor reward-contingent behavior, and when the ACC detects non-conducive behavioral deviations (e.g., lapses in attention) the prefrontal cortex is recruited to exercise top-down control to calibrate behavior, by way of increasing LC phasic activity, to maintain optimal performance (Aston-Jones and Cohen, 2005; Sara and Bouret, 2012; Massar et al., 2016; Bier et al., 2019). As engagement in the task continues, the costs associated with optimal performance tend to increase, while the rewards associated with the task tend to decrease due to increased satiety, exposure, or predictability of the rewards, eventually resulting in increased LC tonic activity.

For instance, Massar et al. (2016) found that performance-contingent rewards fostered better performance and longer engagement in a vigilance task. Moreover, pretrial pupil size was also greater for those who received rewards as well, suggesting greater engagement in the task. Similarly, when drivers interacted with a system that gamified driving and rewarded drivers for good performance (e.g., degree of lateral steering control, hazard avoidance, etc.) during a manual drive, subjective fatigue was delayed, standard deviation of lane position and unintentional lane crossings were reduced, drivers were less prone to accidents, and there was better compliance with driving at the speed limit (Bier et al., 2019). Rewards may be introduced to help rebalance a task's utility by fostering motivation and optimizing one's arousal state for the task at hand (Aston-Jones and Cohen, 2005; Boksem and Tops, 2008). Some have even suggested that NE acts as a “network resetting” (Bouret and Sara, 2005) or “circuit-breaking” (Corbetta and Shulman, 2002) signal that reconfigures network connectivity for the purpose of maximizing rewarding outcomes.

The LC-NE system may influence broad network connectivity possibly due to its connections to the frontoparietal control network (FPCN; Sara and Bouret, 2012). According to the global workspace theory, the integration of various neural submodules forms conscious experience and underlies the engagement in various cognitive processes (Baars et al., 2003; Smallwood et al., 2012a). The FPCN is thought to be responsible for housing a “global workspace” that consolidates all cortical communication and facilitates the activity of the most salient submodule. Because of this, the FPCN plays a critical role in not only directing attention, for instance, based on the most dominant submodule, but also maintaining attention as well, be it externally or internally oriented.

Within a vigilance context, two key networks that contribute to the global workspace of the FPCN are the dorsal attention network (DAN) and the default mode network (DMN; Dang et al., 2012). When attention is oriented externally, the FPCN couples with the DAN, while coupling with the DMN when attention is directed internally (Spreng, 2012). The LC-NE may be one of the primary neuromodulatory systems, alongside the ventral tegmental area-dopamine system (VTA-DA), that dictates which network the FPCN couples with by adjusting the gain of neuronal activity (Dang et al., 2012; Ranjbar-Slamloo and Fazlali, 2020).

Neural gain signifies the specificity of functional connectivity and a greater signal-to-noise ratio of strong neuronal communication (Mittner et al., 2016). For instance, when neural gain is low, broad functional connectivity can be observed, with weaker connections becoming more active and therefore competitive with stronger connections. This would translate to greater distractibility and engagement in other tasks (e.g., mind wandering). Conversely, during high neural gain, functional connectivity becomes precise by suppressing weaker connections, while stronger, task-relevant connections remain dominant. While in a state of low arousal, tonic LC activity tends to be high, which in turn reduces neural gain and allows task-unrelated cortical activity (e.g., DMN) to influence the global workspace of the FPCN to facilitate explorative behavior. In contrast, while in a state of optimal arousal, phasic LC activity tends to be high, which increases neural gain, and promotes exploitative behavior by facilitating the coupling of the FPCN and the DAN (Aston-Jones and Cohen, 2005; Bouret and Sara, 2005; Sara and Bouret, 2012; Mittner et al., 2016).

In sum, the LC-NE system is implicated in a wide range of cognitive processes, such as attention, memory, mood, motivation, perception, and arousal. This is likely due to its broad afferent and efferent connections and the fact that it is the primary source of NE in the neocortex (Aston-Jones and Cohen, 2005; Bouret and Sara, 2005; Sara and Bouret, 2012). The LC-NE, receiving input from the ACC and OFC, plays a critical role in optimizing task performance by modulating arousal levels, which in turn broadens or narrows our attentional filter for rewarding outcomes. Within a vigilance context, it can also influence the FPCN by adjusting the neural gain associated with environmental stimuli, thereby facilitating the coupling between the DAN (task-related) or the DMN (task-unrelated). When a driver is tasked with monitoring the automation system, the understimulating nature of the task may induce hypo-arousal by influencing the firing rate of the LC-NE due to an imbalance of the costs for having to maintain vigilance (Körber et al., 2015). Additionally, this imbalance may foster the activity of task-unrelated brain networks and promote engagement in non-driving related tasks such as mind-wandering (Bier et al., 2019). The LC-NE system is a critical component in understanding the cause of low arousal, or passive fatigue. Passive fatigue, however, only addresses one aspect of fatigue; depleted cognitive resources, or active fatigue, can address another.

## Processes Underlying Active Fatigue

Active fatigue differentiates itself from passive fatigue by examining fatigue as a function of cognitive load and time on task (Warm et al., 1996; Grier et al., 2003; Szalma and Claypoole, 2019), as opposed to arousal (e.g., Scerbo, 1998; Hockey, 2011; Langner and Eickhoff, 2013). Most of the explanation regarding the link between active fatigue and its two components are based upon “cognitive resources;” however, despite decades of research it has yet to be objectively, or even collectively, defined (Dehais et al., 2020). Below, the present authors build upon those previous works by describing active fatigue from a more objective perspective—as a function of long-term neuronal potentiation.

It is generally thought that the act of maintaining vigilance over a prolonged period of time is very taxing on cognitive resources (Warm et al., 1996). This idea intuitively makes sense, given that some sort of “cost” is always associated with some sort of activity in the real world (e.g., time, attention, money, etc.). However, “cognitive resources” have remained obtuse and no clear definition has been presented (Dehais et al., 2020). The notion of some “cost” or resource requirement for any cognitive activity, however, should not wholly be discarded. Specifically, in the context of vigilance, a decrement could occur not due to an over-expenditure of cognitive resources *per se*, but rather an overaccumulation of synaptic load. First, however, it is important to clarify the meaning of resource depletion by disentangling correlates of brain activity from indicators of brain metabolism.

Brain activation refers to the changes in blood flow, specifically the arterial oxygen concentration, as reflected by the oxygen extraction fraction (OEF) when using positron emission tomography (PET) or the blood-oxygen-level-dependent (BOLD) signal when using functional magnetic resonance imaging (fMRI). Brain metabolism, on the other hand, refers to the oxidation of glucose, through aerobic glycolysis and oxidative phosphorylation, to create adenosine triphosphate (ATP; Raichle and Gusnard, 2002; Raichle and Mintun, 2006). Simply put, the former refers to oxygen delivery, while the latter refers to oxygen consumption. When researchers observe “brain activity,” oxygen delivery is increased in a local region of the brain, under the assumption that greater neuronal activity occurred in that region; oxygen consumption. However, although it does increase slightly, it does not increase to the same degree (Raichle and Gusnard, 2002). Changes in oxygen delivery in a specific region of the brain does not necessarily entail meaningful changes in oxygen consumption, or energy expenditure. In other words, brain activation and brain metabolism are two distinct processes. They are related, yet independent.

One of the assumptions underlying BOLD signals, for instance, is that local blood-flow changes supply the necessary ingredients (oxygen and glucose) to create ATP to fuel task-induced brain activity. Raichle and Mintun (2006), however, argued that this assumption is somewhat misguided, if not wholly incorrect. They note that the genesis of this assumption stems from research showing the relatively strong correlations of single-unit recordings, multiunit recordings, and local field potentials with changes in fMRI BOLD signals. However, this only demonstrates a correlational relationship between local blood-flow changes and neuronal activity - it does not demonstrate a causal relationship. Moreover, single-unit and multiunit recordings represent different aspects of neuronal activity compared to local field potentials. Specifically, the former refers to the spiking activity of neurons, or the output, while the latter refers to the membrane currents of neurons, or the input. Therefore, it is unclear why and how changes in local blood-flow are related to neuronal activity. In addition, there is usually a lag time of 4–6 s regarding task-induced changes in BOLD signals. The brain, as Raichle and Mintun argued, would not depend on such a slow process to provide the necessary moment-to-moment prerequisites for brain activity. Instead, it would be more efficient to extract more of the oxygen that is already circulating in the blood and to use the glucose that is already stored in the glycogen of astrocytes, suggesting that the brain does not necessarily depend on increases in local blood-flow to fuel brain activity.

Interestingly, there are no significant changes in whole-brain blood flow due to the engagement of a goal-directed task, with only negligible (≤5%) differences in local blood-flow (Raichle and Gusnard, 2002). Moreover, the brain's metabolism, or energy budget, also remains strikingly constant as well, again irrespective of engaging in a task or passively at rest, suggesting that the traditional paradigm of resource depletion may not be the most appropriate conceptualization of active fatigue. When taking into account that the vast majority of the brain's metabolism is allocated toward maintaining proper excitatory and inhibitory synaptic activity (Raichle and Mintun, 2006), investigating synaptic processes, specifically its homeostasis, could help illuminate the fatiguing nature of vigilance tasks.

Every animal—be it terrestrial, oceanic, or avian—engages in sleep to regulate synaptic activity that took place during wakefulness (Vyazovskiy and Harris, 2013). With prolonged wakefulness, cognitive and physical deficits, and even death, inevitably occur (Wang et al., 2011). The synaptic homeostasis hypothesis (Tononi and Cirelli, 2006) can help explain the rebalancing that takes place as a function of both wakefulness and sleep, and it makes four claims regarding how the brain achieves equilibrium: (1) synaptic potentiation occurs predominantly during active wakefulness. During wakefulness, plastic changes, specifically long-term potentiation, occur due to the presynaptic firing and postsynaptic depolarization associated with a broad range of neuronal activity. Evidence comes from the increases in synaptic density, due to long-term potentiation, that are typically found when animals are in stimulus-enriched environments. (2) Slow wave activity (SWA) regulates the homeostasis of synaptic potentiation. Predominantly observed in non-rapid eye movement (NREM) sleep, SWA are spontaneous oscillations consisting of low frequency (<1 Hz), high amplitude (>140 μv) sequences of synchronized depolarized up-phases (On-periods) and hyperpolarized down-phases (Off-periods; Van Someren et al., 2011). Delta waves (1–4 Hz) with amplitudes ranging from 75 to 140 μv have also been thought to reflect slow wave activity as well but with less cortical synchronization. A localized concentration of SWA is commonly observed in a location-dependent manner based on the area of synaptic potentiation that occurred during wake in both cortical and subcortical regions (Vyazovskiy and Harris, 2013; Bernardi et al., 2015; Quercia et al., 2018; Andrillon et al., 2019). (3) Slow wave activity facilitates homeostasis primarily through synaptic downscaling. Synaptic weight, or load, is accumulated onto neurons as a function of use-dependent, long-term potentiation. Slow wave activity acts to proportionally downscale, or decrease, the synaptic weight of neurons engaged in long-term potentiation, thereby resetting the net load accumulated during wake. Specifically, the magnitude of SWA tends to correlate with how long one prolongs wakefulness and exponentially decreases as one remains asleep (Vyazovskiy et al., 2011). Finally, (4) synaptic downscaling is one of the ultimate causes of cognitive restoration. Evidence for this claim comes from the uncompromising, and intrusive, occurrences of SWA during prolonged wakefulness, such as micro-sleeps (Dinges, 1995). Moreover, irregularities in SWA have been connected to various mental disorders, such as depression, and sleep-related disorders, such as insomnia (Tononi and Cirelli, 2006). In fact, Vyazovskiy and Harris (2013) suggest not only does the resetting of neuronal firing rates plays a critical role in cognitive restoration, but that SWA may be a self-defense countermeasure that acts as cellular maintenance to prevent unnecessary, long-term damage (e.g., excessive oxidative stress and damage to DNA, proteins, and lipids). For instance, in the face of cognitively demanding tasks, neurons will maintain high synaptic activity for optimal performance until neuronal fatigue sets in, as indicated by periods of neuronal silencing. As neurons continue to fatigue, SWA becomes more intrusive in terms of frequency and spatial location, translating into greater decrements in performance (Van Someren et al., 2011; Andrillon et al., 2019).

To summarize, active fatigue may not be best described as an expenditure of finite cognitive resources *per se*, because the brain's energy budget remains relatively constant, irrespective of task engagement (Raichle and Gusnard, 2002; Raichle and Mintun, 2006). Instead, we propose that active fatigue may be more accurately thought of as long-term potentiation of neurons inducing a high synaptic load (Tononi and Cirelli, 2006). Evidence for this stems from the observation that the vast majority of the brain's energy budget is allocated toward regulating excitatory and inhibitory synaptic activity. When neuronal fatigue occurs, the brain will engage in sleep-related processes, such as SWA, to compensate for high synaptic load (Vyazovskiy and Harris, 2013). For instance, Bernardi et al. (2015) demonstrated indicators of SWA in visuomotor and executive functioning areas when participants were controlling a driving simulator for a prolonged time, and the presence of SWA was associated with poorer performance. Sleep-related processes during cognitively demanding tasks could result in not only poorer performance but also promote engagement in unrelated tasks, such as mind-wandering (Andrillon et al., 2019). Alternating SWA in different neural locations (e.g., DAN vs. DMN), for example, may represent a major source of naturally occurring cognitive restoration and homeostasis.

## Circadian Processes Subserve Passive and Active Fatigue

Independent of task characteristics, arousal can also fluctuate based on our circadian rhythm (Carrier and Monk, 2000; Aston-Jones et al., 2001). Our circadian rhythm, or clock system, is primarily controlled by a central clock—the suprachiasmatic nucleus of the hypothalamus (SCN)—with influences coming from a myriad of peripheral clocks found in all our tissue (Nicolaides et al., 2014). The SCN plays a significant role in our sleep-wake cycle by increasing alertness through arousal regulation (Aston-Jones et al., 2001). Specifically, circadian variations in arousal are influenced by a circuit consisting of the SCN, the dorsomedial nucleus of the hypothalamus (DMH), and the locus coeruleus (LC). Light/dark cues from the environment modulate SCN activity. The SCN sends projections to the DMH, which acts a relay for the LC, and the LC, in turn, elicits a NE response, thereby influencing arousal. The SCN-DMH-LC circuit may partially explain the ultradian time-of-day variations found in a variety of cognitive processes, such as fatigue, alertness, and memory (Carrier and Monk, 2000; Van Dongen and Dinges, 2000; Aston-Jones et al., 2007). Moreover, NE in blood plasma also exhibits a circadian rhythm with numerous ultradian peaks throughout wakefulness (Sowers and Vlachakis, 1984). Interestingly, these NE rhythms also closely align with time-of-day variations found in mind-wandering rates (Smith et al., 2018), subjective alertness, arousal ratings, and driving performance (Lenné et al., 1997). Not only do circadian processes independently influence passive fatigue (Carrier and Monk, 2000; Aston-Jones et al., 2001), they can also independently influence active fatigue as well (Bernardi et al., 2015). Before describing these influences, it is important to acknowledge the blurred boundaries between wakefulness and sleep.

Wakefulness and sleep are generally thought of as two distinct states, but electrophysiological evidence indicates they can occur simultaneously. Wakefulness can generally be thought of as a state in which most of the brain is active, while in a sleep state, most of the brain is quiescent. But, cortical and subcortical regions can briefly engage in sleep-related processes while other regions remain “awake,” referred to as local sleep (Vyazovskiy and Harris, 2013). Local sleep is defined as the “transient, regional neurophysiological state showing a mixture of features characteristic of (i) wakefulness and sleep, (ii) different sleep stages (NREM and REM sleep), or (iii) different sleep depths (light or deep sleep)” (Andrillon et al., 2019, p. 2). One indicator of local sleep is the presence of high-amplitude, slow oscillations (i.e., SWA). As previously described, slow oscillations are a series of synchronized neuronal activity (On-periods) and neuronal silencing (Off-periods) among different cortical areas that can occur locally in terms of both time and space (Vyazovskiy et al., 2011).

The occurrence of local sleep, as indexed by SWA, is dependent on two factors: time spent awake and use-dependent activity (Vyazovskiy et al., 2011; Bernardi et al., 2015; Quercia et al., 2018; Andrillon et al., 2019). In terms of time, local sleep tends to be more frequent as sleep pressure, or the need to sleep, builds. Conversely, local sleep tends to be less frequent the longer one remains in sleep (Van Someren et al., 2011). For instance, local sleep rarely occurs in the first few hours after wake, but gradually appears as one remains awake (Vyazovskiy et al., 2011). Microsleeps are an extreme example of local sleep occurring during wakefulness (Dinges, 1995; Andrillon et al., 2019) and have been associated with poorer driving (Boyle et al., 2008) and increased accident rates (Sirois et al., 2009). In NREM sleep, SWA tends to be most prominent in frontal and parietal regions (Vyazovskiy et al., 2011). Though both areas generally tend to exhibit synchronized SWA concurrently (global), they can occasionally—particularly during wakefulness—occur independently (local) as well. As the number of occurrences of SWA increases, more areas of the brain engage in SWA (Andrillon et al., 2019). Drastic changes in various neuromodulators could also be indicators of local sleep as well.

From a circadian perspective, we exhibit three vigilance states: wakefulness, NREM sleep, and REM sleep (Aston-Jones et al., 2001, 2007). These three states are distinguishable based on various electrophysiological, physiological, and neurochemical metrics. During wakefulness, high-frequency, low-amplitude activities are common, specifically in the gamma (30–60 Hz) and beta (20–30 Hz) frequencies. We also generally have higher heart rates and heart rate variability compared to the other vigilance states. During NREM sleep, low-frequency, high-amplitude activities become common, such as frequent occurrences of sleep spindles (12–15 Hz), delta waves (1–4 Hz), and slow waves (<1 Hz). Heart rate and brain temperature also decrease during this NREM state as well, compared to wakefulness. Finally, in REM sleep, we exhibit similar brain patterns as wakefulness (high-frequency, low-amplitude signals). In fact, REM sleep is also referred to as paradoxical sleep due to its striking resemblance to wakefulness. It is impossible to distinguish REM sleep from wakefulness when only observing one's electrophysiology. The differences between REM sleep and wakefulness can be found when examining one's physiology, specifically transient paralysis in the muscles accompanied by rapid changes in body temperature during REM sleep. There are neurochemical differences between the three vigilance states. Significant changes in tonic firing of NE occur such that the firing rate tends to be highest during wakefulness, then decreases dramatically during NREM sleep, and becomes almost completely dormant during REM sleep. Specifically, changes in NE occur prior to the transitions between vigilance states (Aston-Jones et al., 2007).

To summarize, passive and active fatigue are distinct constructs, yet they are related in that they independently operate under a circadian rhythm (Carrier and Monk, 2000; Aston-Jones et al., 2001). The SCN-DMH-LC circuit regulates our arousal based on light/dark cues from the environment, and because of this, arousal fluctuates throughout the day due to our sleep-wake cycle (Aston-Jones et al., 2001). Sleep and wakefulness, however, are not mutually exclusive states. Specifically, cortical regions engage in sleep-related processes (i.e., local sleep) based on the degree of neuronal activity that has occurred in that region during wakefulness (Quercia et al., 2018; Andrillon et al., 2019). In this way, circadian processes sit at the junction between passive and active fatigue, and this triumvirate could explain how and why fatigue occurs.

## Conclusion

Driver distraction and drowsiness, or fatigue more broadly, have continued to be some of the leading causes for motor vehicle crashes, and these crashes have continued to be one of the leading causes of death in the United States. Understanding the mechanisms of cause-and-effects for why fatigue occurs is critical to improving road safety. Fatigue is a multidimensional construct that may have at least three components: passive, active, and sleep-related fatigue. Although these components have different causes, they are interrelated in that they influence each other. Passive fatigue tends to occur due to various exogenous characteristics inducing hypo-arousal and it can promote engagement in non-driving related tasks to alleviate boredom. Active fatigue occurs as a function of long-term neuronal potentiation in which specific brain regions will engage in sleep-related processes to avoid unnecessary long-term damage due to prolonged activity. Despite these disparate processes, they are both influenced by our circadian rhythm. In other words, time-of-day moderates how passive and active fatigue occurs.

Finally, we would be remiss for not also mentioning alternative theories, outside of the under- and overload theories described above, whose shoulders' this article stands upon. Two, in particular, warrant specific mentioning. First is Hockey's (2011) motivational control theory that provides a compelling case for the dynamic interplay between fatigue and recovery, and second are the works of Hancock (2013, 2017) who elegantly described the weaknesses of previous theories and discusses the impact those weaknesses have had in our understanding of vigilance. The interested reader is highly encouraged to read these original works to challenge their assumptions regarding the latent-variable construct of fatigue.

It is our hope that this review increases general understanding of the specific processes that subserve different aspects of fatigue. And further, that understanding the mechanisms underlying passive and active fatigue, and the influence of circadian rhythms on each will facilitate the development of effective driver fatigue countermeasures for each type.

## Author Contributions

All authors contributed to the article and approved the submitted version.

## Conflict of Interest

The authors declare that the research was conducted in the absence of any commercial or financial relationships that could be construed as a potential conflict of interest.

## Publisher's Note

All claims expressed in this article are solely those of the authors and do not necessarily represent those of their affiliated organizations, or those of the publisher, the editors and the reviewers. Any product that may be evaluated in this article, or claim that may be made by its manufacturer, is not guaranteed or endorsed by the publisher.
